# Protective and Therapeutic Efficacy of *Mycobacterium smegmatis* Expressing HBHA-hIL12 Fusion Protein against *Mycobacterium tuberculosis* in Mice

**DOI:** 10.1371/journal.pone.0031908

**Published:** 2012-02-21

**Authors:** Shanmin Zhao, Yong Zhao, Fengfeng Mao, Caiqin Zhang, Bing Bai, Hai Zhang, Changhong Shi, Zhikai Xu

**Affiliations:** 1 Division of Infection and Immunology, Laboratory Animals Center, Fourth Military Medical University, Xi'an, Shaanxi Province, China; 2 Department of Microbiology, Fourth Military Medical University, Xi'an, Shaanxi Province, China; University of Delhi, India

## Abstract

Tuberculosis (TB) remains a major worldwide health problem. The only vaccine against TB, *Mycobacterium bovis Bacille Calmette-Guerin* (BCG), has demonstrated relatively low efficacy and does not provide satisfactory protection against the disease. More efficient vaccines and improved therapies are urgently needed to decrease the worldwide spread and burden of TB, and use of a viable, metabolizing mycobacteria vaccine may be a promising strategy against the disease. Here, we constructed a recombinant *Mycobacterium smegmatis* (rMS) strain expressing a fusion protein of heparin-binding hemagglutinin (HBHA) and human interleukin 12 (hIL-12). Immune responses induced by the rMS in mice and protection against *Mycobacterium tuberculosis* (MTB) were investigated. Administration of this novel rMS enhanced Th1-type cellular responses (IFN-γ and IL-2) in mice and reduced bacterial burden in lungs as well as that achieved by BCG vaccination. Meanwhile, the bacteria load in *M. tuberculosis* infected mice treated with the rMS vaccine also was significantly reduced. In conclusion, the rMS strain expressing the HBHA and human IL-12 fusion protein enhanced immunogencity by improving the Th1-type response against TB, and the protective effect was equivalent to that of the conventional BCG vaccine in mice. Furthermore, it could decrease bacterial load and alleviate histopathological damage in lungs of *M. tuberculosis* infected mice.

## Introduction

*Mycobacterium bovis Bacille Calmette-Guerin* (BCG), a live, attenuated mycobacterial strain first used in humans in 1921 is still currently the only vaccine available against tuberculosis (TB) [1], but its protection is extremely variable. While effective against the severe forms of the disease in children, BCG displays limited effects on adult pulmonary TB and transmission of the causative agent, *Mycobacterium tuberculosis* (MTB) [2]. Hence, improved vaccines against TB are desperately needed. *Mycobacterium smegmatis* is a rapidly growing saprophyte, able to propagate one generation every 1–3 h. It is non-pathogenic and commensal in humans and can act as a powerful cellular immune adjuvant [3], [4]. *M. smegmatis* also has a number of properties that renders it an effective vaccine vector. This fast-growing *Mycobacterium* is unable to arrest phagolysosome maturation and cannot evade intracellular killing [4], [5], [6]. Moreover, its rapid clearance by the host differs from that of *M. tuberculosis* or even the vaccine strain BCG [7]. *M. smegmatis* can activate dendritic cells and induce CD8-mediated immune responses, and immunization with recombinant *M. smegmatis* has been shown to generate more durable memory T cells as compared to intramuscular DNA vaccination [8], [9], [10]. These observations encourage further development of mycobacteria as efficient recombinant vaccine delivery vectors.

Aside from having an efficient delivery vector, the choice of an immunogenic target antigen is also important for developing a successful vaccine. The heparin-binding hemagglutinin (HBHA) is a mycobacterial cell surface protein that mediates adhesion to epithelial cells and that has been implicated in the dissemination of *M. tuberculosis* from the site of primary infection [11]. The lymphocytes from healthy human individuals infected with *M. tuberculosis* produce high levels of HBHA-specific interferon-γ (IFN-γ). Protective immunity induced by methylated HBHA is comparable to that afforded by vaccination with BCG, and DNA vaccination with the HBHA gene has resulted in both HBHA-specific antibodies and IFN-γ production [12], [13]. Recombinant HBHA which has no methylation produced in *Escherichia coli* is not immunogenic. Methylation of HBHA is required for the full immunological properties of the protein [14]. It has been proved that HBHA produced in recombinant *M. smegmatis* (rMS) can express the immunogenic methylated form of HBHA [15].

Mycobacterial infections lead to the activation of innate immunity, followed by the induction of the Th1 T cell subset, which is thought to be influenced by IL-12 in an antigen-specific fashion [16]. IL-12 is a novel potential cytokine immunotherapy for the treatment of *Mycobacterium tuberculosis* infection. It has been proved IL-12 cound stimulate lymphocytes to produce Th1 cytokines and enhance both innate and cellular immunity in many ways against intracellular pathogens [17]. Okada M [18] reported that DNA vaccine expressing mycobacterial heat shock protein 65 and IL-12 exerted strong therapeutic efficacy (100% survival and augmentation of immune responses) in the TB-infected monkeys.

In order to further enhance the immunogenicity of HBHA and hIL-12 against *M. tuberculosis* infection, we generate a multivalent, vectored vaccine candidate utilizing the *M. smegmatis* strain to tandemly express HBHA and hIL-12. Subcutaneous immunization of this recombinant *M. smegmatis* vaccine (rMS) is performed to evaluate its efficacy and protective immune responses against *M. tuberculosis* in mice. Furthermore, the mouse infection with *M. tuberculosis* is also used to evaluate the therapeutic efficacy of the rMS.

## Results

### Expression of HBHA-hIL12 fusion protein by rMS

**Figure 1:**
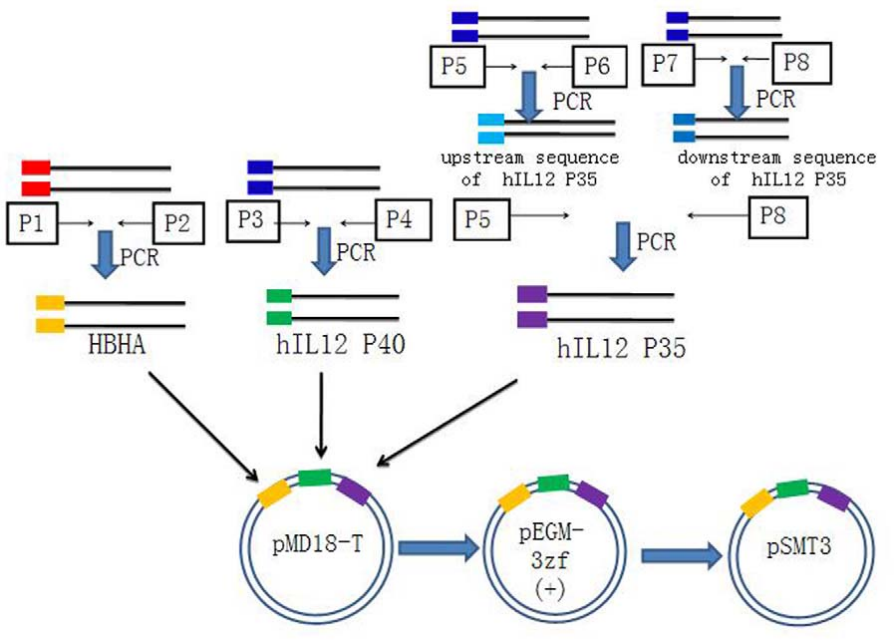
Basic gap-repair cloning procedure. The HBHA gene was PCR cloned from *M. tuberculosis* genomic DNA using the primer pair: p1 and p2. hIL12 P40 was cloned from the cDNA using the primer pair: p3 and p4. The 542 base pair upstream sequence of hIL12 P35 was amplified with primers p5 and p6, and the 220 base pair downstream sequence of hIL12 P35 was extracted using the primer p7 and p8. The two PCR products then then used as templates to amplify the full hIL12 P35 gene using the primer pair: p5 and p8. The PCR product was cloned into the pMD18-T vector for sequencing and subsequently transferred to the cloning vector pEGM-3zf(+) for digestion with the appropriate restriction endonucleases for insertion into the pSMT3 construct.

**Figure 2:**
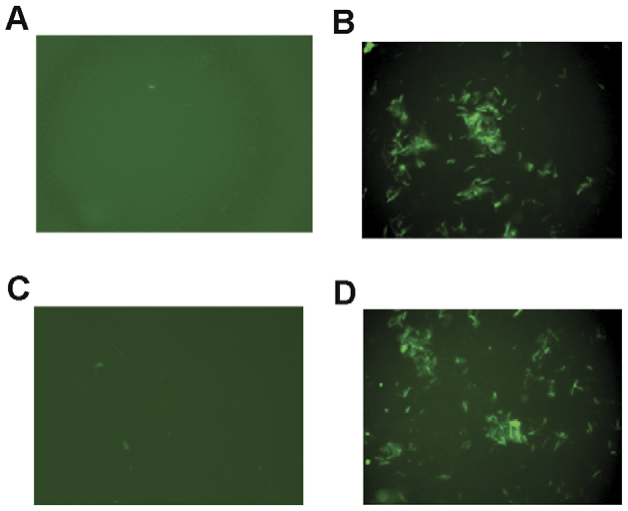
Immunofluorescence detection of HBHA-hIL12 fusion protein expressed in rMS (1000×). (A) Binding of *M. smegmatis* (negative control) with anti-HBHA mAb. (B) Binding of rMS with anti-HBHA mAb. (C) Binding of *M. smegmatis* with anti-hIL12 mAb. (D) Binding of rMS with anti-hIL12 mAb.

**Figure 3:**
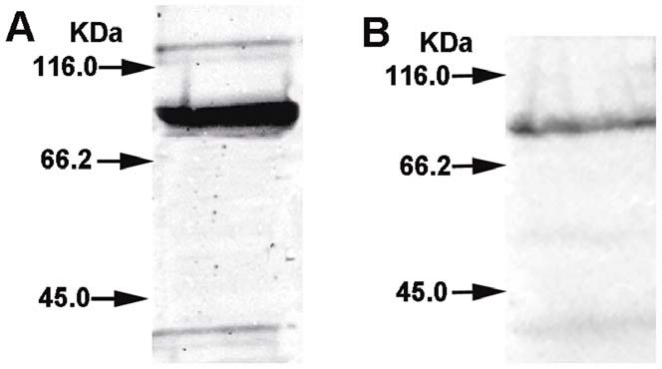
Expression of HBHA-hIL12 fusion protein in the rMS by Western blot analysis. (A) The expressed fusion protein was recognized using anti-HBHA mAb. (B) The expressed fusion protein was recognized using anti-hIL12 mAb.

In order to create a recombinant *M. smegmatis* strain to express a fusion protein of HBHA and hIL12, the expression vector was first constructed by cloning the HBHA and hIL12 genes as described in Methods (Fig. 1). Sequence of all the resulting PCR products (not shown) were similar to that reported in GeneBank. The HBHA-hIL12 expression cassette including an encoded flexible linker was then cloned into pSMT3 by utilizing the *Bam*H I and *Hin*d III restriction endonucleases. The positive rMS stains were screened on 7H10 agar plates containing OADC and hygromycin, and confirmed by PCR (not shown). The HBHA-hIL12 fusion protein expressed in rMS was detected by staining with antibodies against HBHA and hIL12 separately. Due to the high fluorescence signals, rMS could easily be detected under a standard fluorescence microscope (Fig. 2). Western blot analysis of the recombinant *M. smegmatis* revealed an expression band at a size of approximately 86 kDa (Fig. 3), which matched the combined molecular weights of HBHA (28 kDa) and hIL-12 (58 kDa). These results confirmed that the HBHA-hIL12 fusion protein was efficiently expressed in rMS.

### Growth rates of *M. smegmatis* and rMS

**Figure 4:**
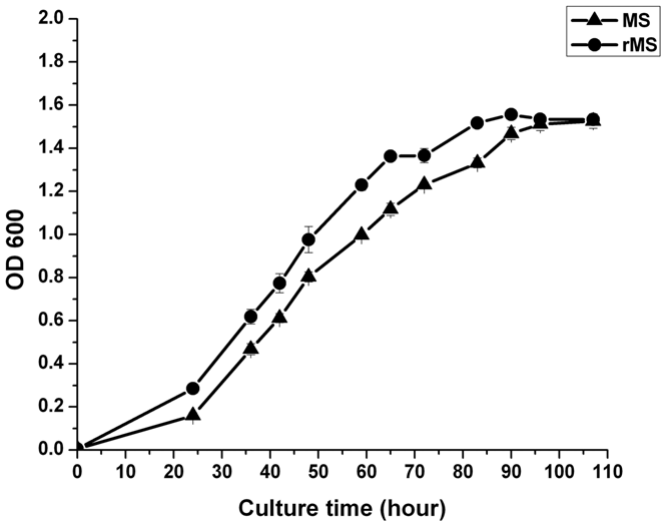
Growth curves of *M. smegmatis* and rMS. Bacteria populations were monitored by optical density at 600 nm. Data are shown as mean ± SD of values from four experiments.

The growth patterns of *M. smegmatis* and rMS were determined over the course of 107 h of culture. OD values of *M. smegmatis* and rMS were observed a similarly consistent trend, entered the plateau phase at the same time (Fig. 4). *M. smegmatis* and rMS strains showed no significant differences in proliferation characteristics..

### IFN-γ ELISPOT responses to TB antigens in splenocytes of immunized mice

**Figure 5:**
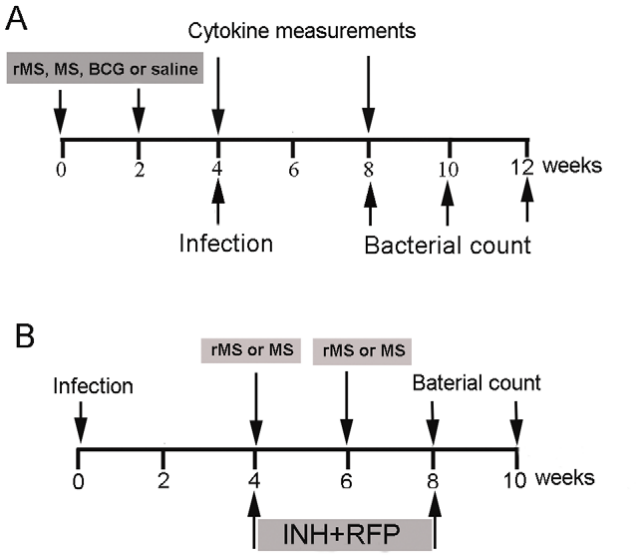
Schedule of treatments and sampling. (A) rMS, *M. smegmatis* or BCG was given two times with a 2-week interval. Serum was obtained from immunized mice at 2 and 6 weeks after the second immunization. The mice were injected with the H37Rv strain via the tail vein at 2 weeks after the second immunization. Determination of bacterial burden and histopathology were performed at 4, 6 and 8 weeks post-challenge. (B) Mice were given NIH+RFP in the drinking water from 4 to 8 weeks post-infection (upper panel). Determination of bacterial burden and histopathology were performed at 8 and 10 weeks post-infection.

**Figure 6:**
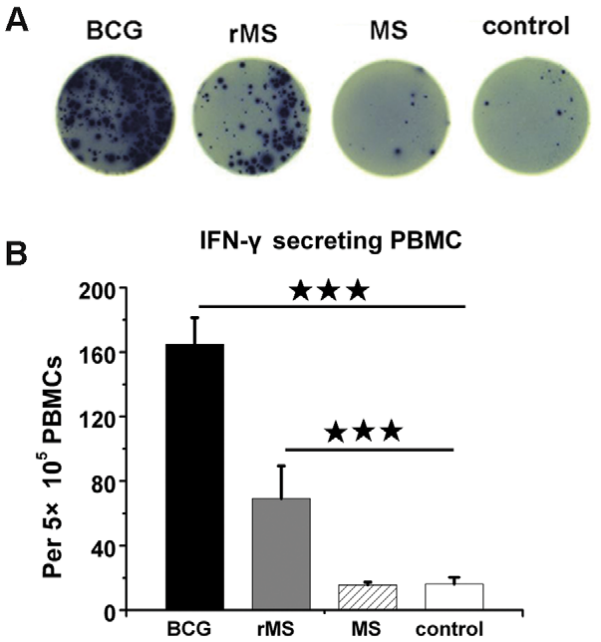
Detection of IFN-γ producing splenocytes by ELISPOT. (A) Photographs of representative wells. (B) Bars representing the mean ± SEM of SFUs. A significant difference was found between the IFN-γ secretion from splenocytes of the rMS group and the BCG group and that from the saline group. No significant difference was found between the saline group and the *M. smegmatis* group.

Mice were inoculated twice at 2 weeks apart with *M. smegmatis*, rMS, BCG or saline as described in Methods (Fig. 5A). Two weeks after the final vaccination, the frequency of antigen-specific IFN-γ cells in the splenocytes of each group was assessed by mouse ELISPOT assays. Significantly increased frequencies of IFN-γ secreting cells were observed in splenocytes of groups vaccinated with rMS or BCG compared to the saline group. By contrast, no significant difference was found between the saline group and the *M. smegmatis* group (Fig. 6).

### Serum cytokine responses to immunization

**Figure 7:**
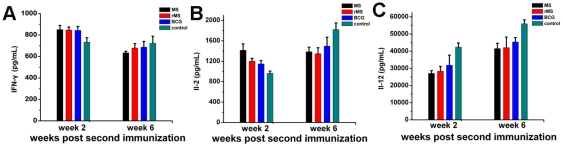
Th1 cytokine induction post-challenge. Total levels (pg/mL) of Th1 cytokines, (A) IFN-γ, (B) IL-2, and (C) IL-12 measured in the serum of rMS, *M. smegmatis*, BCG or saline immunized mice 2 weeks and 6 weeks following second immunization. Results are expressed as mean ± SEM of 3 animals per group.

In examining the serum cytokine profile elicited by rMS, *M. smegmatis* and BCG, we observed increases in serum IFN-γ and IL-2 at 2 weeks following the second immunization compared to the saline group. Meanwhile, significantly diminished levels of IFN-γ and IL-2 were found in the rMS, *M. smegmatis* and BCG immunized mice 6 weeks following immunization compared to the saline group (Fig. 7A and B). However, the levels of IL-12 were higher in the saline group than in the rMS, *M. smegmatis* and even BCG groups at two measured time points (Fig. 7C).

### CD4+ and CD8+ T cell phenotypes in PBMCs of immunized mice

**Figure 8:**
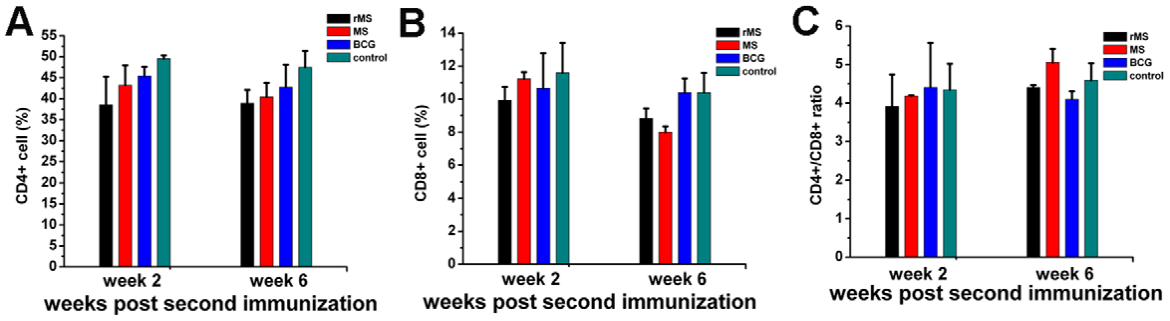
Percentages of CD4+ and CD8+ T cells in PBMCs from immunized mice. Groups of mice were vaccinated twice 2 weeks apart subcutaneously with rMS, *M. smegmatis*, BCG or saline. Three animals from each group were euthanized 2 weeks and 6 weeks following second immunization. Results are expressed as mean ± SEM of 3 animals per group.

Cellular composition of PBMCs was assessed by 2-color flow cytometry. The total CD4^+^ T cell numbers decreased following BCG vaccination, although it was not statistically significant at 2 weeks (*P* = 0.13) and 6 weeks (*P* = 0.28) following vaccination. The total CD8^+^ T cell numbers were not significantly different between the BCG group and the saline group after 2 and 6 weeks (*P* = 0.59 and *P* = 1, respectively). Compared to these results with BCG vaccination, similar trends in the total number of CD4^+^ and CD8^+^ T cells were observed following rMS vaccination, The total CD4^+^ T cell number of the rMS group was lowest among the 4 groups at both 2 and 6 weeks post-vaccination (Fig. 8A). A decrease was found in the total CD8+ T-cell number in the *M. smegmatis* group when comparing the 2 week and 6 week time points (*P*<0.001) (Fig. 8B).

### Protective effects of the rMS vaccine

**Figure 9:**
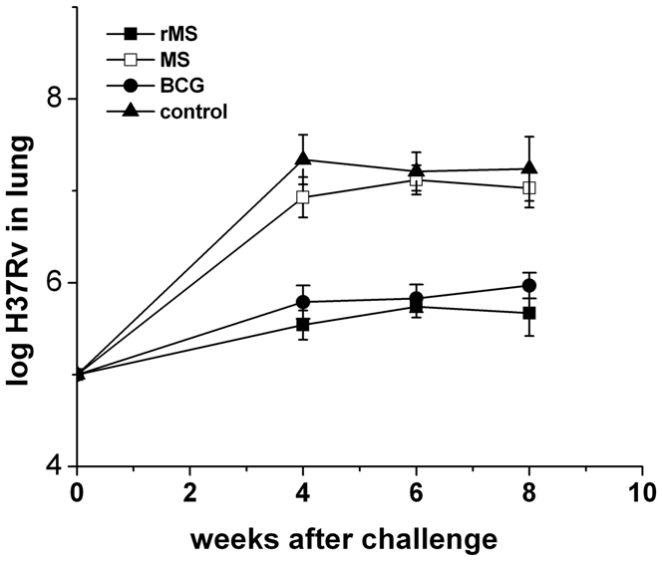
Assessment of protection in immunized mice. Growth of virulent H37Rv in the lungs of mice immunized with rMS, *M. smegmatis*, BCG or saline was assessed 4, 6, and 8 weeks after challenge. Results are expressed as mean ± SEM of 3 animals per group.

**Figure 10:**
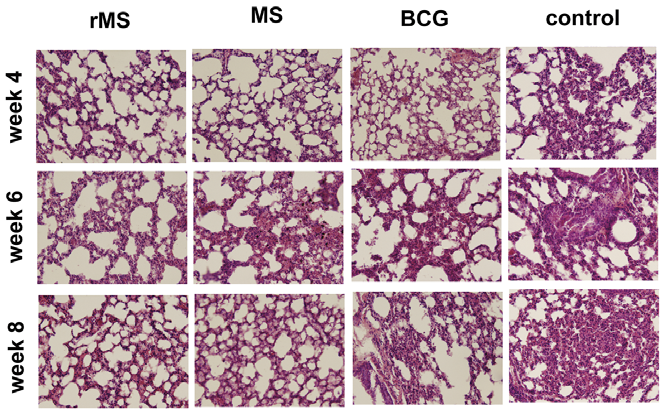
Histopathology of lung tissue after challenge. Sectioned lung tissue from naive and immunized mice taken 4, 6 and 8 weeks after virulent challenge (all panels 400× magnification, H&E stained).

A treatment protocol was designed (Fig. 5B). Four weeks after the immunized mice were infected with the *M. tuberculosis* H37Rv strain the bacterial loads were 5.54±0.16, 6.93±0.22, 5.79±0.18 and 7.34±0.27 per lung in the rMS, *M. smegmatis*, BCG and saline immunized mice, respectively (Fig. 9). During the initial phase of infection, mice immunized with the rMS showed robust inhibition of growth of virulent *M. tuberculosis*. A similar trend in reduction of bacteria load compared to saline immunized mice was observed at 6 and 8 weeks following challenge. Considerably lower loads of the virulent strain were observed in the lungs of rMS immunized mice after 4, 6 and 8 weeks of infection compared to the saline control group (Fig. 9). This data revealed that the protective effect of the rMS was equivalent to that provided by BCG (*P*>0.05) over the course of infection. In the *M. smegmatis* group, we observed a reduced bacillary load in the lungs compared to the saline group at 4 weeks post *M. tuberculosis* infection (*P*<0.05), but the bacteria loads were not significantly different between two groups after 6 and 8 weeks of infection (*P*>0.05). Analysis of lung histopathology in mice immunized with rMS revealed less lung consolidation compared to the control mice (saline-treated) (Fig. 10). Based on the observed pathological changes, the protective effect provided by rMS in the lungs was apparently equivalent to that by the BCG vaccine.

### Therapeutic effects of the rMS vaccine

**Figure 11:**
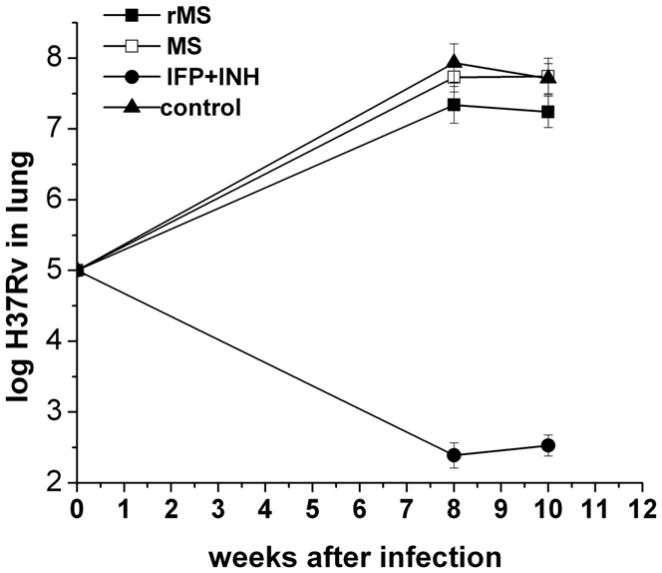
Therapeutic effects of rMS, *M. smegmatis* or INH+RFP on bacterial burden of lungs from *M. tuberculosis*-infected mice. Four weeks after infection with H37Rv, mice were treated with rMS or *M. smegmatis* two times with a 2-week interval, and lung mycobacterial loads were determined 8 and 10 weeks after challenge. INH (54.25 mg/L) co-administered with RFP (52.5 mg/L) was delivered in drinking water for 4 weeks from 4 weeks after infection. Results are expressed as mean ± SEM of 3 animals per group.

**Figure 12:**
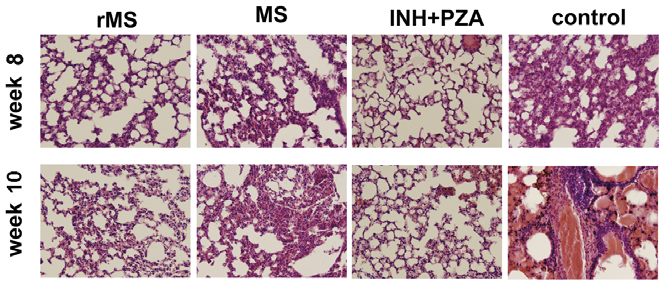
Effect of rMS, *M. smegmatis* or INH+RFP on histopathology of lungs from *M. tuberculosis*-infected mice. Sectioned lung tissue from rMS, *M. smegmatis* or INH+RFP treated mice 8 and 10 weeks after virulent challenge (all panels 400× magnification, H&E stained).

Mice infected with the *M. tuberculosis* H37Rv strain were treated with either rMS or *M. smegmatis*, and the therapeutic effects were determined by the bacteria load in the lung. The decrease in bacterial burden in mice that received rMS correlated with a decrease in lung consolidation and number of pathological lesions when compared to the saline treated group (*P*<0.05) at the two measured time points (Fig. 11). A significant reduction in the reactivation of *M. tuberculosis* was observed in the lungs of mice treated with INH+RFP. These results indicated that rMS played a role in the inhibition of *M. tuberculosis* infection; however, treatment with the rMS vaccine was significantly less effective than treatment with INH+RFP. As shown in Figure 12, extensive lymphocytic infiltration and aggregation of interstitial mononuclear cells could be seen in the pulmonary alveoli of mice treated with two doses of rMS. The *M. smegmatis*-treated mice showed more lymphocytes accumulating around the bronchii (Fig. 12) and had significantly greater pathological damage similar to the saline controls and more polymorphonuclear leukocytes than observed in lungs of rMS-treated mice. In the group treated with NIH+RFP, lesions in the lungs were slight, and much less aggregation of lymphocytes could be seen than in the other three groups (Fig. 12).

## Discussion

*Mycobacterium smegmatis* is an attenuated species of *Mycobacterium* that has been previously used as a carrier of vaccine antigens against tuberculosis with ambiguous results [19]. However, it has shown promise as an effective vaccine vector to deliver cytokines that can skew Th1 immunity in mice [16]. We previously evaluated a recombinant vaccine prepared from *M. smegmatis* expressing a fusion of early secreted antigenic target 6-kDa antigen (ESAT6) and culture filtrate protein 10 (CFP10) [20]. After *M. tuberculosis* challenge in mice, a dramatic reduction in bacteria load in the lungs was observed for the mice immunized with the rMS (rMS-e6c10). The protective efficacies of rMS-e6c10 and BCG vaccination were found to be similar based on measures of *M. tuberculosis* burden and lung pathology.

We have constructed a vector pQE80-HBHA in *E. coli*, but recombinant HBHA produced in *E. coli* does not effectively protect mice from *M. tuberculosis* challenge as reported. HBHA produced in *E. coli* is not immunogenic. The native HBHA protein contains 20–26 methyl groups on residues 159–199. These methyl groups are not present in the recombinant form of HBHA produced by *E. coli*, and methylation of HBHA is required for the full immunological properties of the protein [13], [21]. Giovanni Delogu *et al.* [22] had constructed a recombinant *M. smegmatis* strain expressing a histidine-tagged recombinant HBHA protein from *M. tuberculosis* (rHBHAms). The methylation pattern of rHBHAms was similar to that observed for nHBHA (rHBHAms ≈16 methyl groups vs. ≈23 methyl groups in nHBHA), and this partial methylation was shown to be sufficient to rescue the immunological properties of HBHA as shown in humoral response studies [15]. They showed the methylated HBHA of *M. tuberculosis* produced in *M. smegmatis* was antigenic and potentially useful to exclude active TB in a T-cell based in vitro system. Then we constructed a vector pSMT3 expressing HBHA protein in *M. smegmatis* (rMS-HBHA). But the protection of this vaccine was significantly less effective than that of rMS-e6c10 which we constructed. The recombinant *M. smegmatis* vaccine (rMS-HBHA) needs to further enhance its immunogenicity. Interleukin 12 (IL-12) plays an important role in anti-intracellular pathogens by augmenting innate and cellular immunity in many ways. It is essential to the generation of a protective immune response to *M. tuberculosis* mainly by the induction of IFN-γ expression and the activation of antigen-specific lymphocytes which could form a protective granuloma [23], [24]. Yang C *et al* [24] had constructed the eukaryotic co-expression plasmid encoding human GLS and murine IL-12, transformed this plasmid into *M. smegmatis*. This rMS had immunotherapeutic effects associated with a switch to the Th1 response and the antibacterial activity of GLS. So we constructed recombinant *M. smegmatis* expressing the IL12 and HBHA fusion protein in order to further improve the immunotherapy effects of rMS on the *M. tuberculosis* infection and confirm its capable of stimulating host specific immune responses against *M. tuberculosis*.

To ensure that the HBHA and IL-12 fusion protein had sufficient space for correct folding was important, we introduced a 48 bp sequence encoding a peptide linker into the forward primer of the hIL-12 gene. This commonly used flexible linker consists of hydrophibic glycine and serine resides and allows two fusion proteins to correctly fold without impacting the biological activity of each protein [25]. The immunofluorescence detection and Western blot analysis of rMS showed that the fusion protein reacted not only with anti-HBHA mAb but also with anti-human IL-12 mAb, indicating that the expressed product of the HBHA–IL-12 fusion gene in eukaryotic cells had intact binding sites for both HBHA and hIL-12 specific antibodies.

An optimized vaccine protocol, in which recombinant *M. smegmatis* is administered followed by boosting, may result in an appreciable increase in its protective efficacy. Cayabyab *et al.* [9] constructed a recombinant *M. smegmatis* strain expressing the human deficiency virus gp120 envelope protein. Mice were inoculated twice with an interval of 10 weeks. The peak responses after the two inoculations were not greater than those seen following a single inoculation, but the responses in the boosted mice remained detectable even 1 year following the initial immunization. Their results suggest that recombinant *M. smegmatis* may be useful as a priming vector in prime/boost vaccine regimens.

In the current study, groups of mice were vaccinated 2 times at 2 weeks apart in a homologous prime-boost schedule. While antigen specific cellular responses was achieved as determined by IFN-γ ELISPOTs, FACS analysis showed that the total CD4^+^ and CD8^+^ cells were not increased in the BCG group compared to the saline group. In the study by Harari *et al.* [14], *M. tuberculosis*-specific CD4^+^ T cell responses in a representative subject with latent *M. tuberculosis* infection were mostly (>70%) polyfunctional, i.e., producing IFN-γ, IL-2 and TNF-α, while a representative subject with active TB disease showed a dominant (>70% of CD4^+^ T cells) TNF-α–only response. Chen *et al.* [26] showed that the number of CD4^+^ CD25^+^ FoxP3^+^ Treg is increased in the blood of active TB patients. Foxp3^+^ CD25^+^ CD4^+^ natural Tregs suppress the proliferation of naive T cells and their differentiation to effector T cells *in vivo*. They can also suppress effector activities of differentiated CD4^+^ and CD8^+^ T cells [27]. BCG-induced immunity is perhaps better described in terms of the T cell subset patterns, such as the proportion of CD4^+^ T cells producing cytokines (e.g., IFN-γ, IL-2 and TNF-α) and even of CD4^+^ CD25^+^ FoxP3^+^ Tregs. It may be the reason that a decreased number of CD4+ cells being observed after vaccination with recombinant *M. smegmatis* in contrast with observation of an increased number of TNF-α producing cells. BCG-induced T cell is perhaps just a part of the T cell subset patterns, not all T cell. In sum, changes of the total CD4+ or CD8+ T cell numbers may be not reflect protective efficacy, and antigen-specific T cells would be a better indicator of protective efficacy than the total number of CD4^+^ and CD8^+^ cells.

Both IFN-γ and IL-2 are not only important indices for the Th1 cell immune response, but also play an important role in the regulation of anti-mycobacterial immune responses by triggering the activation and proliferation of CD4+ T cells [28]. The IFN-γ and IL-2 were found to increase in the blood of mice immunized with rMS when assessed 2 weeks after the second immunization, compared to mice that had received *M. smegmatis* or saline. And we observed significantly increased frequencies of IFN-γ secreting cells in PBMCs of mice vaccinated with rMS and BCG but not with *M. smegmatis* compared to the saline group 2 weeks after the second immunization. At 6 weeks following immunization the levels of IFN-γ and IL-2 were diminished significantly in the *M. smegmatis*/rMS immunized mice. An increase in these Th1 cytokines always suggested a higher ability to induce a greater protective effect [28], [29], [30]. It may preferentially promote a T-helper type 1 (Th1) cell mediated response during the primary phase of infection. Though quantitative measurement of cytokines is widely used to assess the ability of a vaccine to induce an immune response, it is not always indicative of resulting protection [31], [32]. Genetic or environmental factors may influence the cytokine profile of mycobacteria specific T cells responses to vaccination [33]. Lalor *et al.* reported that Malawian infants have been shown to generate cytokine responses following BCG vaccination, but the cytokine profile is different from that in the UK [34]. The number of multifunctional T cells making IFN-γ, TNF-α, and interleukin 2 (IL-2) did not correlate with protection against disease in South Africa [33], [35]. Therefore, we considered that measurements of cytokines could be useful to evaluate a candidate vaccine but are not always proper indicator of vaccine efficacy. Further evaluations of the vaccine should be done, especially counting the bacterial load [CFU] in target organs and evaluating changes in pathology.

The bacillary load in lungs is the most important parameter used for the evaluation of protective efficacy following challenge with *M. tuberculosis*. So we examined the bacteria load in the lung at three measured time points. The results shown that the protection of rMS is at least as well as the control BCG vaccine in terms of reducing load of the H37Rv strain and lung histopathology. After the mice infected with the H37Rv were treated with rMS, the bacterial numbers in the lungs of the mice were reduced significantly. Previous research has shown that wild-type *M. smegmatis* is unable to provide protection against challenge with *M. tuberculosis* in mice [36]. However, our data showed that a reduced bacillary load in the lungs of *M. smegmatis*- immunized mice at 4 weeks post *M. tuberculosis* infection compared to the saline group (*P<0.05*), but the bacteria loads were not significantly different between two groups after 6 and 8 weeks of infection (*P>0.05*), which may be due to *M. smegmatis* providing protection at the initial phase of infection, or different immunization strategies.

Immunotherapy that modulates or enhances the host immune response to *M. tuberculosis* has proven to be an effective method for treatment of TB [37]. It has been a long-held contention that specific resistance to *M. tuberculosis* infection generated by a live vector is better than that of non-living vaccines [38]. DNA vaccines that were being investigated in mice for prophylactic use against TB were soon found also to be surprisingly effective in treatment against established infection [39]. Our past study demonstrated that the HSP65-IL-2-DNA vaccine enhances the immunogenicity and protective as well as therapeutic effects of the HSP65-DNA vaccine against TB in mice by improving the Th1-type response [25]. However, immunity against *M. tuberculosis* is more efficiently generated by viable, metabolizing mycobacteria than by DNA vectors. Although BCG can activate specific immune responses against tuberculosis and is the most successful immunotherapeutic reagent for solid malignancy currently, it cannot be directly used as a therapeutic vaccine for tuberculosis patients, especially those in an immune deficiency state because of its significant toxicity. In contrast, *M. smegmatis* is a fast growing, low virulence strain that has been tested experimentally as a vaccine candidate for *M. tuberculosis* [40], as well as an alternative gene expression system for BCG or *M. tuberculosis* [41]. The recombinant *M. smegmatis* carrying IL-12 and GLS can induce an efficient Th1 protective immunity response, including a high level of IFN-γ and IL-12 similar to the BCG strain [16].

Therefore, we investigated whether the rMS could be effective as a treatment against established infection. After administration of the rMS in TB infected mice, lung bacterial load and lymphocytic infiltration decreased significantly, although not to the levels achieved by the chemotherapy drugs, compared to the saline controls. Long-term treatment with anti-tuberculosis drugs INH+RFP has been shown to result in a significant reduction in lung inflammation and accelerated resolution of lung pathology. However, a long treatment course, along with the side effects, often results in treatment failure due to drug resistance and financial problems. Of great concern, there are now extensively drug-resistant (XDR) strains emerging, which are resistant to essentially all of the currently available drugs. Thus, obtaining novel, shorter treatment regimens is an important objective of anti-tuberculosis drug development. Many studies have indicated that a DNA vaccine combined with chemotherapy improves bacillary clearance and results in smaller granulomas and less lung pathological damage, compared to treatment with chemotherapy alone [37], [42], [43], [44], [45]. In the next work, we will evaluate the effectiveness of combination treatment with the rMS and chemotherapeutic drugs.

## Materials and Methods

### Ethics statement

All animal protocols have been reviewed and approved by the Institutional Animal Care and Use Committee of the Fourth Military Medical University (ID11013).

### Bacterial strains and *in vitro* growth conditions

The *M. tuberculosis* H37Rv strain was obtained from the Institute of Drug and Biological Products Checking (Beijing, China). The BCG vaccine strain (Denmark strain) was obtained from the Lanzhou Bioethical Production Institute (Lanzhou, China). The human peripheral blood was obtained from the Department of Immunology of the Fourth Military Medical University (Xi'an, China) [46]. Mycobacterial cultures were grown in Middlebrook 7H9 broth (Difco Laboratories, Detroit, MI) supplemented with 0.5% glycerol and 10% BBL Middlebrook ADC enrichment (Beckton Dickinson Biosciences, Oxford, UK). For determining the colony forming units (CFU), bacilli were grown on Middlebrook 7H10 agar (Difco) supplemented with 0.5% glycerol and 10% BBL Middlebrook OADC enrichment (Beckton Dickinson).

### Plasmid and strain construction

The HBHA gene was cloned from the *M. tuberculosis* genomic DNA (NC_000962.2) via PCR using the primer pair: p1 (5′-GCGGATCCATGGCTGAAAACTCGAACATTG-3′) and p2 (5′-ATGTCGACCTTCTGGGTGACCTTCTTG-3′). Total RNA generated from human peripheral blood was extracted using ISOGEN-LS according to manufacturer′s protocol (Nippon Gene Co, Tokyo, Japan). First-strand complementary DNA (cDNA) was synthesized by reverse transcriptase using the RNA as template. hIL12 P40 (AF180563.1) was cloned from the cDNA using the primer pair: p3 (5′-GCGTCGACGGTGGCTCAGGTGGCTCCGGTGGAGGCGGAAGCGGCGGTGGAGGATCAATGTGTCACCAGCAGTTG-3′) and p4 (5′-ATGCATGCACTGCAGGGCACAGATGC-3′). The 542 base pairs upstream of hIL12 P35 (AF180562.1) was extracted using the primer pair p5 (5′-ATGCATGCGTTCCTGGAGTAGGGGTACCTGGGGTGGGCATGTGGCCCCCTGGGTCAG-3′) and p6 (5′-GGGTCCATCAGAAGTTTTGCATTC-3′); and the 220 base pairs downstream sequence was amplified with P7(5′-CAAAACTTCTGATGGACCCTAAGAGGC-3′) and p8 (5′-GACAAGCTTTTAGGAAGCATTCAGATAGC-3′). The two PCR products were then used to amplify the full hIL12 P35 gene using the primer pair: p5 and p8. After confirming by sequencing (AuGCT Biotechnology, Beijing, China), the PCR product was cloned into the pMD18-T vector and analyzed by 1% (w/v) agarose gel electrophoresis for 60 min at 100 V in TAE 1× electrophoresis buffer, visualized using 0.06 µg ml^−1^ of ethidium bromide (BioRad, Madrid, Spain) and photographed under UV light. Using the cloned HBHA and hIL12 genes, the HBHA-hIL12 fusion expression cassette was generated by the gap repair method as above with a linker designed to maintain the correct biological activity of both HBHA and hIL-12. A verified clone with the correct sequence (AuGCT Biotechnology) was transferred into a cloning vector pEGM-3zf(+), then cut with the appropriate restriction endonucleases and inserted in the *Escherichia coli-BCG* shuttle plasmid pSMT3 construct. The resulting plasmid was electroporated into *M. smegmatis* using standard techniques [47] to generate the recombinant *M. smegmatis* strain expressing the HBHA-hIL12 fusion protein.

### Determination of antigen expression

The rMS strain was grown in 7H9/ADC (7H9 containing 10% ADC) until mid-log phase and blocked with 10% bovine serum albumin. Glass slides were coated with rMS and probed with a 1∶500 dilution of anti-hIL12 monoclonal antibody (Santa Cruz Biotechnology, Santa Cruz, CA, USA) for 1 h at 37°C. The slides were washed several times with PBS and then incubated with a fluorescein isothiocyanate (FITC)-labeled goat anti-mouse IgG secondary antibody (Kirkegaard & Perry Laboratories, Inc., Gaithersburg, MD) in 1% Evans Blue (as a general protein counterstain). After repeated washes, the slides were observed at 1000× magnification under a fluorescence microscope. The expression of the HBHA protein in rMS was verified using the same method. The fusion protein expression was also identified by western-blot using anti-HBHA mAb (BEI Resources, VA, USA) and anti-hIL12 mAb respectively.

### Determination of growth rates of *M. smegmatis* and rMS

*M. smegmatis* and rMS were grown in Middlebrook 7H9 medium supplemented with 0.5% glycerol and 10% ADC. Two hundred microliters of the cultures were added to conical tubes containing 100 ml culture medium and incubated with shaking at 200 rpm at 37°C. Each culture was sampled (2 ml) at 0, 24, 36, 42, 48, 59, 65, 72, 83, 90, 96 and 107 h of incubation for OD measurement at 600 nm. All tests were repeated 4 times.

### Experimental animals

Six- to eight-week-old male BALB/c mice, provided by the laboratory animal center of the Fourth Military Medical University, were randomly divided into four groups (15 per group). Fifteen mice per group were immunized subcutaneously by inoculating 0.2 ml of a suspension containing approximately 1×10^5^ CFU of *M. smegmatis*, rMS or BCG, and an equal volume of normal saline was used in the negative control group. The immunization was repeated 2 weeks later. Two weeks after the second immunization, 6 mice in each group were used for analysis of lymphocyte proliferation and production of IFN-γ, IL-2 and IL-12. The other 9 mice in each group were retained for the infection experiment with *M. tuberculosis* virulent strain H37Rv. All animal protocols have been reviewed and approved by the Institutional Animal Care and Use Committee of the Fourth Military Medical University (ID11013).

### IFN-γ ELISPOT assays of splenocytes

Two weeks after the second vaccination, mice were sacrificed and their spleens removed aseptically in RPMI-1640 medium containing 10% fetal calf serum (FCS). Spleens were gently ground through a 70-µm cell strainer, and then single-cell suspensions were prepared by density gradient centrifugation using Lympholyte-M (Cedarlane Labs, Burlington, NC, USA). Cells were counted and plated at 5×10^5^ cells per well in the same medium as described above. Following the manufacturer's instructions, the mouse interferon IFN-γ ELISPOT kit (Mabtech, Nacka, Sweden) was used to determine microscopically the relative number of IFN-γ-secreting splenocytes, each represesented by individual spots on the developed membrane. Spleen cells from all groups were plated in triplicate at cells per well in 100 µl medium and then stimulated with the purified protein derivative (PPD) for 48 h at 37°C. PPD (10 µg/ml) was also used to stimulate the cells acquired from each group. The cells were removed and plates were subsequently washed and incubated for 2 h at 37°C with a biotin-conjugated anti-mouse IFN-γ secondary antibody. The filters were developed with prepared Streptavidin-HRP solution at 100 µl per well and incubated for 1 h at room temperature. Spots were counted using an automated ELISPOT reader (Champ II ELISPOT reader system [Sage Creation]).

### Serum cytokine measurements

Serum was obtained from immunized mice at 2 and 6 weeks after the second immunization and evaluated for their IFN-γ, IL-2 and IL-12 content. The assays were performed according to the manufacturer's guidelines of the mouse IFN-γ, IL-2 and IL-12 ELISA kits obtained from Jingmei Company (Shenzhen, China).

### Analysis of peripheral blood CD4 and CD8 T cell subsets

Two and six weeks after the second immunization procedure, antigen-reactive T cells with CD4 and CD8 phenotypes were purified from peripheral blood mononuclear cells (PBMCs) in the mice using FITC-labeled rat anti-mouse CD4 and RPE-labeled rat anti-mouse CD8 antibodies, respectively. Double-color flow cytometry was used to determine the changes in lymphocyte subsets. Ten thousand cells were analyzed from each sample, and data from three different samples from individual mice were used in the statistical analysis. All reagents for cell isolation and antibodies employed in FACS analysis were obtained from BD Pharmingen.

### *M. tuberculosis* infection test

Two weeks after the second immunization, 9 mice from each group were used for challenged with *M. tuberculosis*. The mice were injected with the H37Rv strain via the tail vein at a dose of 10^5^ CFU/mouse, and the lungs of 3 mice per time point were removed aseptically for analysis at 4, 6 and 8 weeks post-challenge.

### Treatment of *M. tuberculosis* infected mice

Six- to eight-week-old male BALB/c mice were infected with the H37Rv strain via the tail vein at a dose of 10^5^ CFU/mouse. Four weeks after infection, the mice were randomly divided into four groups (6 per group). Two groups were treated with 0.2 ml of a suspension containing approximately 1×10^5^ CFU of *M. smegmatis* or rMS. The drug treated group were co-administered isoniazid (INH, 54.25 mg/L) with rifampicin (RFP, 52.5 mg/L) delivered in drinking water for 4 weeks. The fourth group received only saline (negative control). Determination of bacterial burden and histopathology were performed at 8 and 10 weeks post-infection.

### Determination of bacterial burden and histopathology

Lungs of 3 mice per time point were aseptically removed and transferred to a 15 ml screw cap tube and homogenized using a Biospec Mini Bead Beater (Bio Spec Products) in a total volume of 1 mL of RPMI-1640 medium. In order to measure the bacterial burden of the challenge strain in immunized animals, diluted organ homogenates were plated onto Middlebrook 7H10 Media Agar Plates. Total CFU counts were determined following 3–4 weeks of incubation at 37°C. For histopathological studies, lung tissue samples were obtained from mice at 4, 6, 8 and 10 weeks post-challenge, fixed in formalin, paraffin imbedded, sectioned and stained with hematoxylin and eosin (H&E) for histological observation.

### Statistical analysis

The statistical significance of the differences among the means was assessed by the least significant difference (LSD)-t test. *P* values<0.05 indicated significant differences.
